# JACMP 2020; the meaning of growth

**DOI:** 10.1002/acm2.12999

**Published:** 2020-08-10

**Authors:** Michael Mills

**Affiliations:** ^1^ Department of Radiation Oncology University of Louisville Louisville Kentucky USA

Growth in an academic journal can be measured in any number of ways. Are the articles being downloaded and read? Is it seeing more submissions? More articles published? More citations? A higher Impact Factor? Generally, is it moving in the right direction with respect to its publication space, the quality of its published articles, and the quality of its new submissions? All of these things can indicate the health of a journal, and therefore, from time‐to‐time, it is worthwhile for us to stop and get a feeling for what the numbers provided by our publisher, Wiley, are telling us.

All of the figures and statistics in this article were gleaned from the Wiley Insights web site on July 11, 2020, with the exception of the Impact Factor numbers. All information and data are current as of May 31, 2020.

First, consider JACMP Downloads, of both HTML and .pdf versions of the articles.

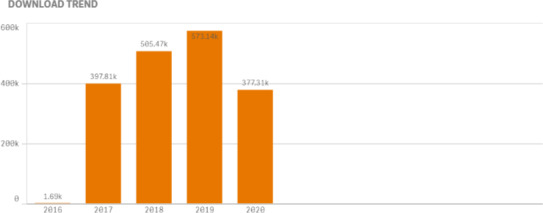

It is encouraging that we have seen almost as many downloads by the end of May 2020 as we did for the entire year in 2017. Download growth is projected to be a 60% increase in 2020 over 2019. However, is there another factor in play?

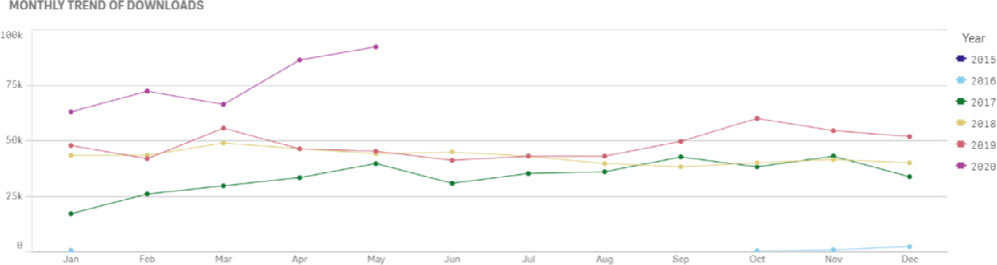

In particular, note the increase in the number of JACMP Downloads in the months of April and May, 2020 over previous years. Could we be seeing the consequence of having large numbers of medical physicists staying at home consequent to the COVID‐19 pandemic, catching up on their research projects and keeping up with the literature? We will certainly revisit this to see if these trends continue.

The Articles Published Trend graph needs a backstory to flesh out its meaning. In March, Wiley became concerned that with the need for many of their staff to work from home, they would be stretched to maintain their high level of publication throughput. For the March, April, May, and June issues, they reduced the number of articles per issue to a manageable count.

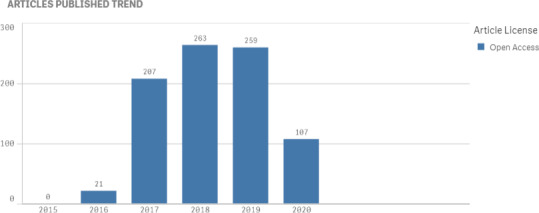

However, by July it became apparent that throughput was not an issue for them and they increased the number of articles per issue for the JACMP to allow it to catch up with a backlog of articles both not yet published in early view and not yet assigned to an issue. We are projecting we should publish over 300 articles in the JACMP in 2020.

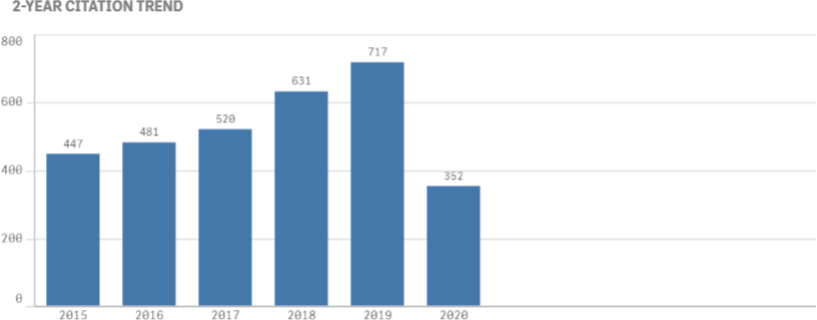

It is gratifying to see the growth in the 2‐year citation trend to be increasing at a greater rate than the number of published articles. This citation rate growth translates to a growth in the Thomson‐ISI 2‐Year Impact Factor for the JACMP. The latest number, published in late June, is for articles published in 2017 and 2018, then cited in 2018 and 2019. 2020 is the first year that all articles reflected by the Impact Factor were published by Wiley.

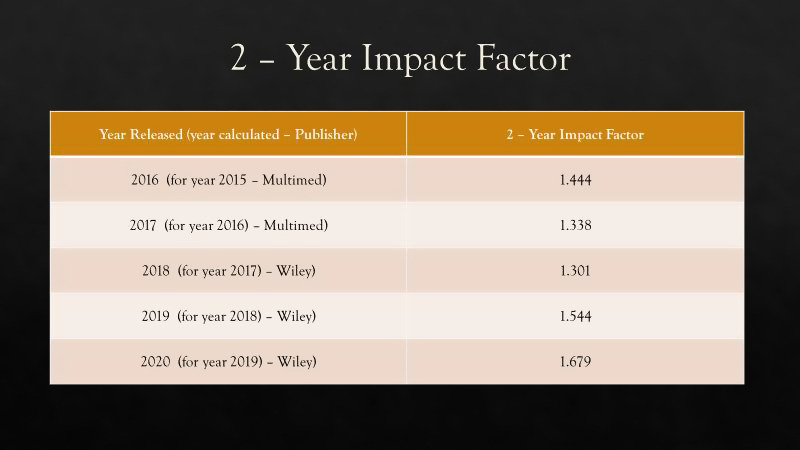

While a journal’s Thomson‐ISI Impact Factor is broadly representative of the quality of its articles and other publications, the JACMP’s steady growth in Impact Factor under Wiley is also reflective of our publisher’s excellent production quality. Additionally, their superior support promoting and marketing the JACMP to the worldwide medical physics and broader radiology communities contributes to the JACMP’s success in these areas.

